# Optimization algorithm for improving the prediction accuracy of API solubility in green solvent

**DOI:** 10.1038/s41598-026-51115-8

**Published:** 2026-06-12

**Authors:** Ali Alasiri, Ahmed A. Lahiq, Abdullah A. Alshehri

**Affiliations:** 1 Department of Pharmaceutics, College of Pharmacy, Najran University, Najran, 11001 Saudi Arabia; 2 Department of Pharmaceutics, College of Pharmacy, Najran University, Najran, 11001 Saudi Arabia; 3 Department of Clinical Pharmacy, College of Pharmacy, Taif University, Taif, 21944 Saudi Arabia

**Keywords:** SC-CO_2_, Drug solubility prediction, Hybrid artificial intelligence, Pareto front analysis, Prediction interval bootstrapping, Thermodynamic modelling, Chemistry, Mathematics and computing

## Abstract

Supercritical carbon dioxide (SC-CO₂) is widely used as an environmentally friendly solvent in pharmaceutical processing, where accurate prediction of drug solubility is essential for efficient formulation design, extraction processes, and process optimization. However, predicting solubility behavior in supercritical systems remains challenging due to the nonlinear interactions between thermodynamic conditions and molecular properties. In this study, a hybrid artificial intelligence framework is developed to predict the solubility of active pharmaceutical ingredients (APIs) in SC-CO₂ using a curated dataset of more than 350 experimentally reported measurements. The proposed framework integrates interpretable deep learning (TabNet) and histogram-based gradient boosting (HGB) with three metaheuristic optimization algorithms, namely the Attack-Leave Optimizer (ALO), Energy Valley Optimizer (EVO), and Botox Optimization Algorithm (BOA), to improve hyperparameter tuning and predictive performance. Model evaluation was conducted using multiple statistical indicators, five-fold cross-validation, prediction interval bootstrapping, and multi-objective Pareto front analysis to assess accuracy and robustness. Among the evaluated configurations, the EVO-tuned TabNet model demonstrated the best predictive performance, achieving a coefficient of determination of $\:{R}^{2}=0.982$along with narrow prediction intervals, indicating strong generalization capability within the studied thermodynamic domain. Statistical analysis using the Kruskal–Wallis test confirmed significant differences between optimizer performances (* p<0.05*). These findings demonstrate that the proposed hybrid pipeline enhances predictive accuracy and interpretability within the thermodynamic domain represented by the compiled dataset. The framework therefore provides a statistically supported computational tool for assisting solvent selection and formulation analysis in supercritical systems, while broader generalization would benefit from future expansion of experimental solubility datasets.

**Supplementary Information:**

The online version contains supplementary material available at 10.1038/s41598-026-51115-8.

## Introduction

Drug solubility is a fundamental property in pharmaceutical sciences because it directly affects bioavailability, formulation strategies, and the overall success rate of drug development. Poor solubility has been repeatedly identified as a major reason for drug discovery failures and attrition during clinical development, as compounds with inadequate solubility often exhibit low absorption and reduced therapeutic effect. For this reason, solubility prediction and measurement have remained central challenges in medicinal chemistry, pharmaceutical analysis, and formulation design across decades of research^1,2^. Traditional solubility determination methods are broadly categorized into thermodynamic and kinetic approaches. Thermodynamic​‍​‌‍​‍‌ methods are used to quantify the maximum amount of solute that can dissolve in a solvent at equilibrium. Thus, they provide a direct measurement of solubility under specified ​‍​‌‍​‍‌conditions. Kinetic processes are directed to find the concentration at which precipitation starts, showing the limit of solubility since the system is still developing and not yet equilibrated^3–5^. Both methods have proven very useful in the discovery of drugs but are both very time-intensive and labor-intensive. They also require an enormous amount of material, which is not feasible for drug candidates that are expensive or hard to come by. Even with increased automation and throughput^6,7^, Classical approaches simply don’t scale well to the requirements of current high-throughput drug pipelines. To address these bottlenecks, computer modeling has come to the rescue as a worthwhile collaborator to experimental methods. Machine learning, in particular, stands out; it leverages existing data on solute-solvent interactions to uncover patterns and predict outcomes under new, untested conditions^8,9^. Initial applications have predominantly concentrated on aqueous solubility, leveraging the extensive availability of experimental datasets to create solvent-specific models with robust predictive capabilities^10^. More recently, there has been a growing interest in extending predictive frameworks beyond a single solvent, incorporating multiple solvents into training datasets to capture broader physicochemical variation and improve generalizability^11,12^. These advances signal a clear trend toward computational strategies becoming increasingly integrated with experimental solubility research^13^. Taken together, these developments underline the expanding role of computational approaches as a complement to traditional solubility measurements in pharmaceutical sciences.

To contextualize the present study within recent advances, Table 1 summarizes representative research efforts on drug solubility prediction using computational and machine learning approaches. The listed studies cover aqueous, mixed, and supercritical solvent systems and collectively illustrate the transition from traditional descriptor-based regressors to more advanced ensemble and deep-learning architectures. In many of these investigations, model performance is further improved through systematic parameter tuning, hybrid modeling strategies, or optimization procedures aimed at enhancing convergence stability and predictive robustness when dealing with nonlinear thermodynamic systems. Each entry outlines the main methodology, data domain, and principal findings to highlight methodological evolution and performance trends across different solubility modeling paradigms^14,15^.

Recent advances in artificial intelligence have further expanded the scope of solubility modeling beyond traditional descriptor-based approaches. Deep learning architectures, including attention-based and graph-based models, have demonstrated improved capability in capturing nonlinear relationships between molecular structure and thermodynamic conditions. In parallel, hybrid frameworks that combine machine learning with optimization algorithms have gained attention for their ability to enhance model calibration and convergence stability. Recent studies have also emphasized uncertainty quantification and model interpretability as critical components for practical deployment, particularly in chemical and pharmaceutical engineering applications.

**Table 1:** Summary of representative computational and machine learning studies on drug solubility prediction.

Refs.	Main methodology	Key conclusions and findings
^16^	Stacked statistical machine learning models on a large aqueous solubility dataset with application domain analysis.	Stacked ensembles of statistical ML outperformed advanced deep learning on the drug domain due to data limits; applicability‑domain analysis enabled confident DrugBank population; experimental solubilities of 10 compounds validated model reliability.
^17^	XGBoost combined with ESP-map and Mordred descriptors.	A simpler XGBoost model with ESP‑map and Mordred descriptors captured key molecular properties and gave strong predictive performance in comparative tests.
^18^	Hybrid COSMO-RS + ML regression workflow.	Combining COSMO‑RS outputs with ML improved solubility predictions versus ML alone, supporting hybrid QM–ML as a viable strategy for aqueous solubility estimation.
^19^	Used ML and deep learning algorithms on curated multi-source datasets, utilizing descriptors and fingerprints.	A model trained on the curated dataset achieved R²≈0.92 and MAE ≈ 0.40 on an external diverse test (Huuskonen), outperforming prior methods on that benchmark.
^20^	Comparison of tree-ensemble models (RF, XGB, LGBM, CatBoost).	Traditional tree‑ensemble algorithms provide satisfactory accuracy and interpretable feature importance for aqueous solubility prediction in drug discovery contexts.
^21^	StackBoost ensemble compared with various boosting and bagging models.	StackBoost achieved highest R² (~ 0.90), RMSE 0.29 and MAE 0.22, outperforming compared ensemble algorithms and showing transferability across datasets.
^22^	NODE and Bayesian neural networks that have undergone hyperparameter optimization.	BNN gave best fit for rivaroxaban in mixed solvents (test R²=0.9926); NODE also performed strongly, indicating advanced ML can capture complex solvent‑composition and temperature effects.
^23^	Extra Trees, RF, and Gradient Boosting for predicting supercritical solubility and density.	For raloxifene solubility ET gave best fit (R²≈0.949, RMSE ≈ 0.41); GB best predicted solvent density (R²≈0.986), indicating ensemble regressors can model supercritical solubility reliably.
^24^	Deep CNN (ResNet50 variants) based on molecular images.	20‑layer ResNet50 produced best results (R²≈0.423, RMSE ≈ 0.678) and high ASP accuracy (90.6%) relative to shallower models and human experts, indicating deep image‑based approaches have utility though numerical gains vs. other methods vary.
^25^	PC-SAFT equation-of-state modeling using fitted parameters.	Parameter‑fitted PC‑SAFT improved extrapolative capability for drug‑polymer solubility predictions, aiding formulation design where experimental data are sparse.

While the studies summarized in Table 1 demonstrate significant progress in solubility prediction using machine learning and thermodynamic modeling, several distinctions can be identified in comparison with the present work. First, a large portion of prior research focuses on aqueous or mixed solvent systems, where relatively extensive datasets and well-established molecular descriptors are available. In contrast, the present study specifically addresses solubility in supercritical CO₂ systems, where experimental data are more limited and the underlying thermodynamic behavior is highly nonlinear. Second, many existing approaches rely either on standalone machine learning models or hybrid combinations with physics-based methods such as COSMO-RS or equation-of-state formulations. The framework proposed here differs by integrating an interpretable deep learning model (TabNet) with an ensemble learning algorithm (HGB) within a unified optimization-driven structure. This combination is designed to simultaneously capture nonlinear feature interactions and maintain stable generalization across structured thermodynamic data. Third, while previous studies primarily emphasize predictive accuracy, the present work incorporates a broader validation strategy, including cross-validation, nonparametric statistical testing, Pareto-based multi-objective optimization, and prediction-interval bootstrapping. This allows model performance to be assessed not only in terms of accuracy but also in terms of stability, uncertainty, and statistical significance. Finally, unlike equation-of-state models that are explicitly grounded in thermodynamic theory or deep learning models trained on large descriptor sets, the proposed approach remains a data-driven framework applied to a limited but diverse experimental dataset. Therefore, its contribution lies in demonstrating how hybrid machine learning and optimization techniques can provide consistent and interpretable predictions within the constrained domain of supercritical solubility data.

Overall, this work aims to improve the reliability and interpretability of solubility prediction for pharmaceutical compounds in SC-CO₂ systems. While the proposed framework demonstrates strong predictive capability within the investigated dataset, methodological rigor is maintained through cross-validation, prediction-interval bootstrapping, and nonparametric statistical testing. Consequently, the reported performance should be interpreted within the thermodynamic domain covered by the compiled experimental data, while further dataset expansion would allow broader generalization across pharmaceutical compounds and operating conditions. The workflow of the present study is organized as follows. First, an experimental dataset of drug solubility in supercritical CO₂ is compiled and preprocessed to ensure consistency and reliability. Second, two predictive models, TabNet and histogram-based gradient boosting (HGB), are developed to capture nonlinear relationships between thermodynamic and physicochemical variables. Third, three metaheuristic optimization algorithms (ALO, EVO, and BOA) are applied for hyperparameter tuning within a cross-validation framework. Fourth, model performance is evaluated using multiple statistical metrics, along with prediction-interval bootstrapping and nonparametric statistical testing. Finally, the results are analyzed through comparative performance assessment, sensitivity analysis, and multi-objective optimization to provide insights into model behavior and practical applicability.

## Dataset

A comprehensive dataset was collected from references^26–35^ to study the solubility behavior of pharmaceutically important compounds in supercritical CO₂ (SC-CO₂). This dataset brings together diverse experimental results, including more than 350 equilibrium data points, each representing carefully controlled measurements of solubility under high-pressure and high-temperature conditions. It covers a wide range of therapeutic classes such as antibiotics, immunosuppressants, antifungals, antivirals, and anticancer agents and includes both small-molecule drugs and large macrolide compounds. This diversity provides a solid foundation for developing data-driven solubility models. The experimental procedures generally used high-pressure equilibrium systems with magnetic stirring to ensure uniform mixing and proper equilibrium establishment.

The solubility measurements were obtained using standard analytical techniques, including gravimetric and spectrophotometric methods, following strict replication and calibration procedures to minimize measurement uncertainty (Table 2). In some experiments, a small amount of cosolvent (ethanol, typically 1–3 mol%) was added to better simulate realistic formulation environments and to observe how solubility changes under near-critical conditions. However, this cosolvent condition is reported only for a subset of the experimental measurements and its concentration range remains relatively narrow. Because such limited variability provides insufficient statistical diversity for reliable feature learning, cosolvent concentration was not incorporated as an input variable in the present modeling framework. Instead, the dataset focuses on four physicochemical descriptors temperature (K), pressure (MPa), molecular weight (g/mol), and melting point (°C) which are consistently reported across all experiments and represent the primary thermodynamic and molecular factors governing solubility behavior in SC-CO₂ systems.

Depending on the compound’s stability and the experimental setup, the temperature in the experiments ranges from about 308 K to 340 K, while the pressure varies from 12 MPa up to over 250 MPa. It encompasses molecules whose molecular weights vary from below 200 g/mol to near 950 g/mol, which indicates that there is a great deal of molecular diversity. Solubility values range over more than two orders of a factor, which highlights important nonlinear relations between molecule structure, thermodynamic properties, and solvent conditions. It provides a chemically varied and finished test standard suitable for testing prediction models of drug solubility in SC-CO₂ systems that has been carefully designed. It is particularly suitable for testing hybrid machine learning-optimization constructs aimed at deciphering complicated solvent-solute interactions and enhancing generalizability of the models.

**Table 2:** Summary of dataset characteristics for drug solubility in SC-CO_2_.

Category	Description	Range/Details
Total observations	Number of experimental equilibrium data points	> 350 measurements
Compound types	Therapeutic and chemical diversity	Antibiotics, immunosuppressants, antifungals, antivirals, anticancer agents
Solvent system	Experimental medium	SC-CO_2_ (with optional 1–3 mol% ethanol cosolvent)
Analytical methods	Solubility quantification techniques	Gravimetric and UV–Vis spectrophotometry (calibrated and replicated)
Input features	Physicochemical parameters	Temperature (K), Pressure (MPa), Molecular Weight (g/mol), Melting Point (°C)
Target variable	Response variable	Solubility (g/L ×10)
Temperature range	Experimental condition range	308–338 K
Pressure range	Experimental condition range	12 – > 300 MPa
Molecular weight range	Molecular diversity among solutes	~ 157–950 g/mol
Melting point range	Crystalline stability variation	146–912 °C
Solubility range	Observed variation in output	0.01 – > 9.0 (g/L ×10)
Experimental setup	Equipment and control method	High-pressure equilibrium cells with magnetic stirring
Dataset purpose	Modeling objective	Predictive modeling of nonlinear solubility behavior in SC-CO₂ systems

Table 3 does something more than just a summary of statistics; it provides a background to select complex hybrid models. The variables have different sizes and are not symmetrical, which suggests that the chemicals are not all the same and that the statistics are not linear. The temperature doesn’t fluctuate significantly, therefore the experimental control is steady. The pressure, on the other hand, varies by two orders of magnitude and is slightly skewed to the right. By​‍​‌‍​‍‌ operating in the mid-range of the pressure-temperature diagram, these characteristics reflect the operational landscape of SC-CO₂ systems, where extreme pressures are not often ​‍​‌‍​‍‌tested. The melting point range and molecular weight vary significantly, including both lightweight and bulky macrolides. This scale variability of physical scale introduces nonlinear molecular structure-solvation energy interactions, which are precisely the sort that ensemble models are best equipped to address. Solubility displays significant asymmetry, characterized by the majority of the molecule exhibiting intermediate values, while a minority presents as extremely soluble outliers. This long-tailed distribution makes sure that the response surface is quite different from a straight line, which is why nonparametric, feature-adaptive learners are needed.‎‎.

**Table 3:** Overview of input features and output variables with their statistical properties.

Features	Units	Statistical properties							
		Min	Max	Median	Mean	Skewness	Kurtosis	Mode	St.Dev
Temperature	(K)	308	338	323	322.9	0.02	-1.31	328	11
Molecular Weight	(g/mol)	12	926.1	315.7	309.3	0.76	-0.12	586.2	266.5
Melting point	(C)	146	914.2	230	332.3	1.37	0.03	146	265.7
Pressure	(MPa)	12	300	30	97.4	0.59	-1.19	128	91
Solubility	(g/L × 10)	0.02	9.05	0.89	1.49	1.90	3.92	0.37	1.7

**Fig. 1:**
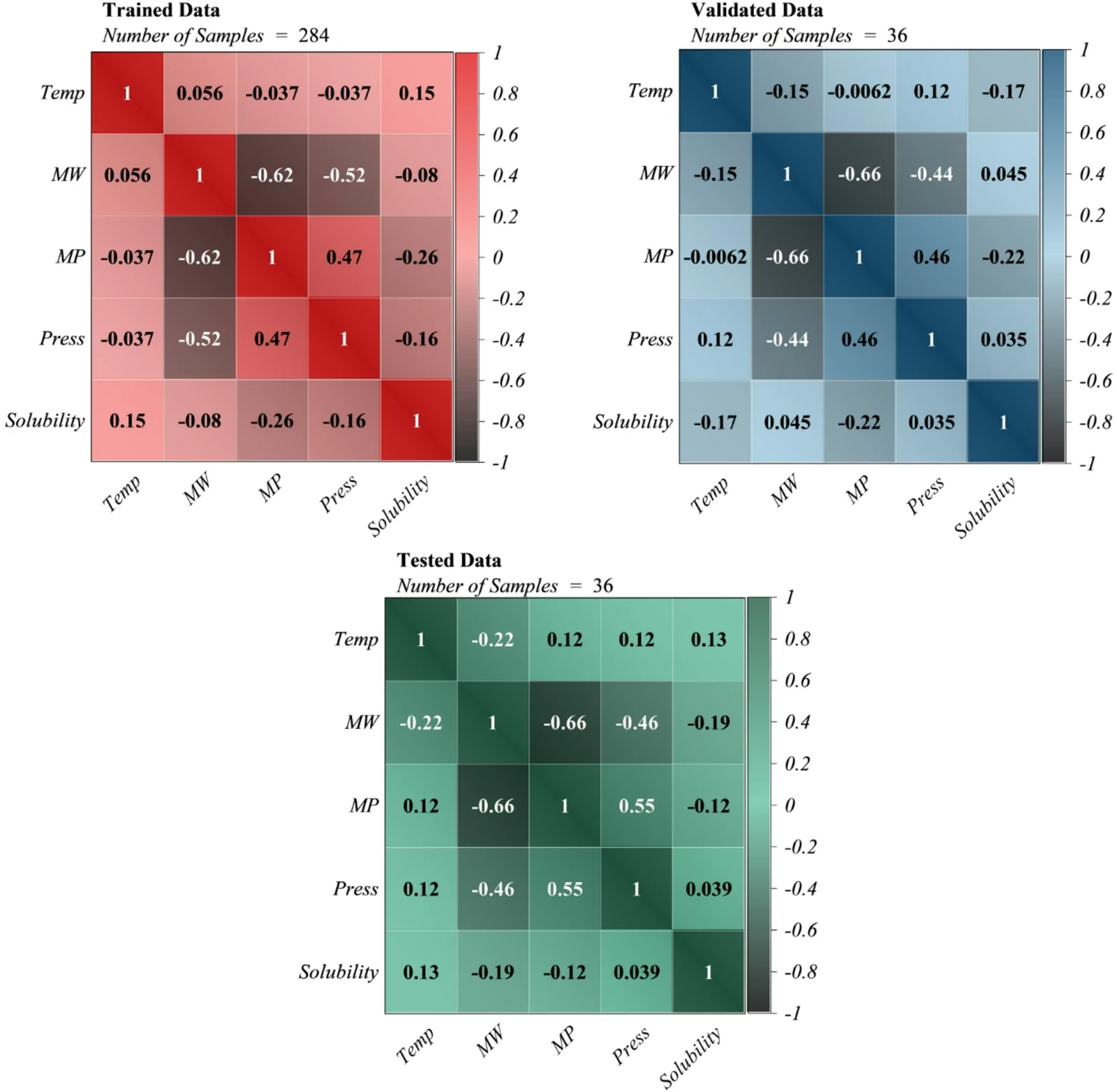
Correlation plot for pairwise relationships and distributions of the dataset variables.

Figure 1 Investigation of pairwise correlations of the primary physicochemical and thermodynamic parameters to reveal intrinsic dependencies that affect drug solubility in supercritical CO₂. The correlation structure remains consistent across the training, validation, and testing subsets, confirming that data partitioning was statistically balanced and free from leakage, an essential prerequisite for generalizable modelling. Temperature and pressure both exhibit mild positive associations with solubility, reflecting the physical principle that higher thermal energy and greater CO₂ density facilitate molecular dispersion and dissolution. In contrast, molecular weight and melting point show moderate negative correlations, indicating that heavier or more crystalline compounds resist solvation due to stronger intermolecular forces and reduced diffusivity. The overall modest correlation magnitudes (< 0.6) suggest that solubility arises from nonlinear, multi-factor interactions rather than from any dominant single variable. This reinforces the need for advanced learning architectures, such as TabNet and HGB, capable of capturing higher-order feature interactions through attention and boosting mechanisms. The consistency of these correlation trends across data partitions further strengthens the dataset’s reliability, ensuring that subsequent optimization with ALO, EVO, and BOA operates on a stable, representative feature landscape.

## Modeling framework

### Choice of models

- TabNet

TabNet temporal attention serves as a selector of intrinsic features that enable interpretation of which physicochemical parameters are the most dominating in drug solubility. Whilst regular networks set static importance, TabNet updates feature importance dynamically over the course of training. This dynamic prioritization proves to be particularly valuable when dealing with the highly divergent and heterogeneous chemical and molecular descriptors that are found in the task of predicting solubility^36^.

- Histogram-Based Gradient Boosting (HGB)

HGB is chosen primarily due to being quick to compute and efficient in capturing complex, nonlinear feature interactions through the use of histogram binning and leaf-wise tree growth. It has a specific strength that makes it best suited to operate alongside tabular pharmaceutical datasets as it prevents overfitting but retains the predictive consistency across a variety of data splits^37,38^.

- Hybrid Justification

Integration of TabNet and HGB brings together the best of both worlds: TabNet offers deep, interpretable feature extraction, and HGB offers aggressive generalization through ensemble learning. With both, the system effectively captures sophisticated chemical relationships and deals with variability present in structured table data. This integration boosts the reliability and accuracy of solubility prediction.

## Hyperparameter optimization algorithms

### Attack–leave optimizer (ALO)

ALO draws inspiration from the dynamics of predator-prey interactions, where attackers pursue while defenders evade within a changing search space. This approach features a dual-phase strategy: it alternates between broad exploration and focused exploitation. Such balance enables effective navigation of complex hyperparameter landscapes, particularly in the context of solubility modeling^39^.

### Energy valley optimizer (EVO)

EVO mimics the energy dissipation and descent behavior of particles in potential valleys, emphasizing rapid convergence and stability. It enhances local refinement in the search space and reduces the chance of premature convergence, ensuring high-fidelity parameter tuning for both TabNet and HGB^40,41^.

### Botox optimization algorithm (BOA)

BOA, inspired by the diffusion and tension adjustment properties of botulinum toxin, focuses on precision**-**driven optimization. It systematically reduces redundant search paths, improving convergence toward the global optimum with minimal computational overhead^42^.

### Application of optimizers

The optimizers are exclusively applied for hyperparameter tuning rather than direct model training. Each optimizer operates within a defined search space tailored to model-specific parameters (as outlined above).

The performance of each optimizer is assessed through:

- Kruskal–Wallis nonparametric testing to statistically compare optimization outcomes.
- Prediction interval bootstrapping to quantify uncertainty and model reliability.
- Pareto front analysis to evaluate the trade-offs between accuracy, interpretability, and computational cost for each optimizer–model pair.

This approach ensures that the final hybrid configuration not only achieves superior predictive accuracy but also maintains statistical significance, reproducibility, and interpretability, essential standards for pharmaceutical modeling studies.

## Performance metrics

To comprehensively evaluate the predictive accuracy, reliability, and robustness of the proposed solubility modeling framework, six statistical indicators were employed: the coefficient of determination (R²), root mean squared error (RMSE), mean absolute percentage error (MAPE), ratio of RMSE to the standard deviation of observations (RSR), uncertainty at the 95% confidence level (U95), and fractional gross error (FGE). Together, these metrics quantify different aspects of model performance, including goodness of fit, absolute and relative error magnitude, predictive uncertainty, and systematic deviation.

- *R² (Coefficient of Determination)* measures the proportion of variance in the observed solubility values that is explained by the predictive model. Values approaching unity indicate that the model reproduces the experimental variability with high fidelity.

1 $$\:{R}^{2}=1-\frac{{\sum\:}_{i=1}^{n}{({y}_{i}-{\widehat{y}}_{i})}^{2}}{{\sum\:}_{i=1}^{n}{({y}_{i}-\stackrel{-}{y})}^{2}}$$

where $\:{y}_{i}$denotes the experimental solubility, $\:{\widehat{y}}_{i}$the predicted value, and $\:\stackrel{\prime }{y}$ the mean of the observed values.

- *RMSE (Root Mean Squared Error)* evaluates the average magnitude of prediction errors and penalizes large deviations more strongly than small ones. Lower values indicate improved predictive accuracy.

2 $$\:RMSE=\sqrt{\frac{1}{n}{\sum\:}_{i=1}^{n}{({y}_{i}-{\widehat{y}}_{i})}^{2}}$$

- *MAPE (Mean Absolute Percentage Error)* expresses prediction error as a percentage of the observed value, allowing performance comparison across different solubility scales.

3 $$\:MAPE=\frac{100}{n}{\sum\:}_{i=1}^{n}\left|\frac{{y}_{i}-{\widehat{y}}_{i}}{{y}_{i}}\right|\:$$

- *RSR (Ratio of RMSE to Standard Deviation of Observations)* normalizes RMSE by the standard deviation of the observed data, producing a dimensionless indicator that facilitates objective comparisons among models.

4 $$\:RSR=\frac{RMSE}{\sqrt{\frac{1}{n-1}{\sum\:}_{i=1}^{n}{({y}_{i}-\stackrel{-}{y})}^{2}}}$$

- $\:{U}_{95}$ *(Uncertainty at 95% Confidence Interval)* represents the predictive uncertainty estimated from the distribution of model residuals and provides an interval-based indicator of reliability.

5 $$\:{U}_{95}=1.96\times\:\sqrt{\frac{1}{n}{\sum\:}_{i=1}^{n}{({y}_{i}-\widehat{{y}_{i}})}^{2}}$$

- *FGE (Fractional Gross Error)* measures the relative deviation between predicted and observed values while maintaining symmetry between overestimation and underestimation.

6 $$\:FGE=\frac{1}{n}{\sum\:}_{i=1}^{n}\left|\frac{{y}_{i}-\widehat{{y}_{i}}}{({y}_{i}+\widehat{{y}_{i}})/2}\right|$$

*Rationale*: Using multiple complementary metrics provides a comprehensive assessment of model performance. R² and RMSE quantify global predictive accuracy, while MAPE and FGE provide interpretable measures of relative error and systematic bias. RSR enables objective comparison across datasets with different variance levels. Finally, U95 introduces an uncertainty-based perspective, reflecting the statistical confidence associated with model predictions. Collectively, these indicators establish a robust framework for evaluating both the accuracy and reliability of predictive models for drug solubility in supercritical CO₂ systems.

## Result and discussion

### Cross-validation reliability

Table 4 shows the five-fold cross-validation results, assessing the reliability and generalizability of the TabNet and HGB models across different data splits. Both models performed well, with high determination coefficients (R² > 0.9) and low RMSE values across all folds, indicating reliable solubility predictions independent of training-test divisions. TabNet consistently outperformed HGB, achieving the lowest RMSE and highest R² in every fold, with approximately 8–10% higher accuracy than HGB. For example, TabNet reached R² = 0.949 and RMSE = 0.427 in Fold 5, demonstrating stable convergence even under challenging data distributions. HGB also performed reliably (R² = 0.917–0.931), though RMSE fluctuated slightly, suggesting limited adaptability to complex feature interactions. The low variation in RMSE and R² between folds (standard deviation RMSE ≈ 0.014 for TabNet, 0.056 for HGB) indicates minimal overfitting and confirms the effectiveness of K-fold validation in reducing sampling bias. In pharmaceutical terms, such reproducibility is essential for regulatory confidence and defensible solubility predictions in formulation development or safety evaluation dossiers according to FDA and EMA guidelines. To further reduce the potential risk of overfitting during hyperparameter optimization, candidate configurations were evaluated across cross-validation folds rather than a single data partition. This strategy improves robustness when working with moderately sized experimental datasets by ensuring that optimized parameters perform consistently across multiple training–validation splits.

**Table 4:** The result of the developed K-Fold.

Models	Indicator	Number of K-Folds				
		*K1*	*K2*	*K3*	*K4*	*K5*
Tabnet	RMSE	0.471	0.458	0.461	0.447	**0.427**
	R^2^	0.940	0.943	0.942	0.946	**0.949**
HGB	RMSE	0.485	0.460	0.595	0.550	**0.450**
	R^2^	0.927	0.930	0.917	0.921	**0.931**

### Optimized hyperparameters and learning dynamics

Table 5 lists the optimized hyperparameters for TabNet- and HGB-based hybrid models, obtained using BOA, ALO, and EVO metaheuristic optimizers. These parameters balance model complexity and generalization, directly affecting predictive stability. For TabNet-based hybrids (TBBO, TBAO, TBEO), BOA selected deeper network architectures that capture strong nonlinear interactions between thermodynamic features. In contrast, ALO and EVO favored sparser settings, promoting smoother convergence and reduced overfitting. HGB-based hybrids (HGBO, HGAO, HGEO) used optimizers to adjust complementary properties, e.g., HGAO applied higher learning rates and shallower trees for faster convergence and regularization, while HGEO used deeper architectures to approximate subtle physicochemical gradients. Each optimizer demonstrates a distinct trade-off between exploration and exploitation, influencing both interpretability and prediction accuracy. In drug research, such hyperparameter tuning enhances generalizability, enabling reproducible solubility predictions across diverse compounds and solvent systems. To ensure reproducibility of the hyperparameter optimization process, predefined search ranges were specified for each model parameter prior to applying the ALO, EVO, and BOA algorithms. These ranges define the exploration boundaries within which the metaheuristic algorithms searched for optimal configurations. The selected intervals were based on commonly used parameter domains reported in the literature for TabNet and Histogram Gradient Boosting models, while allowing sufficient flexibility for the optimization algorithms to identify effective parameter combinations.

**Table 5:** Search space and optimized hyperparameters of the developed hybrid models.

Hybrid models	Hyperparameter	Search range	Optimized value
TBBO	N_d	8–64	33
	N_a	8–64	31
	N_steps	3–10	10
	Gamma	1.0–2.0	2.0
TBAO	N_d	8–64	27
	N_a	8–64	29
	N_steps	3–10	6
	Gamma	1.0–2.0	1.7
TBEO	N_d	8–64	15
	N_a	8–64	15
	N_steps	3–10	4
	Gamma	1.0–2.0	1.5
HGBO	Learning rate	0.01–1.0	0.295
	Max leaf nodes	5–50	13
	Max depth	10–1000	999
	Min samples leaf	1–10	1
	Max bins	64–255	251
HGAO	Learning rate	0.01–1.0	0.602
	Max leaf nodes	5–50	7
	Max depth	10–1000	648
	Min samples leaf	1–10	2
	Max bins	64–255	191
HGEO	Learning rate	0.01–1.0	0.224
	Max leaf nodes	5–50	30
	Max depth	10–1000	346
	Min samples leaf	1–10	2
	Max bins	64–255	187

### Convergence behavior of hybrid models

**Fig. 2:**
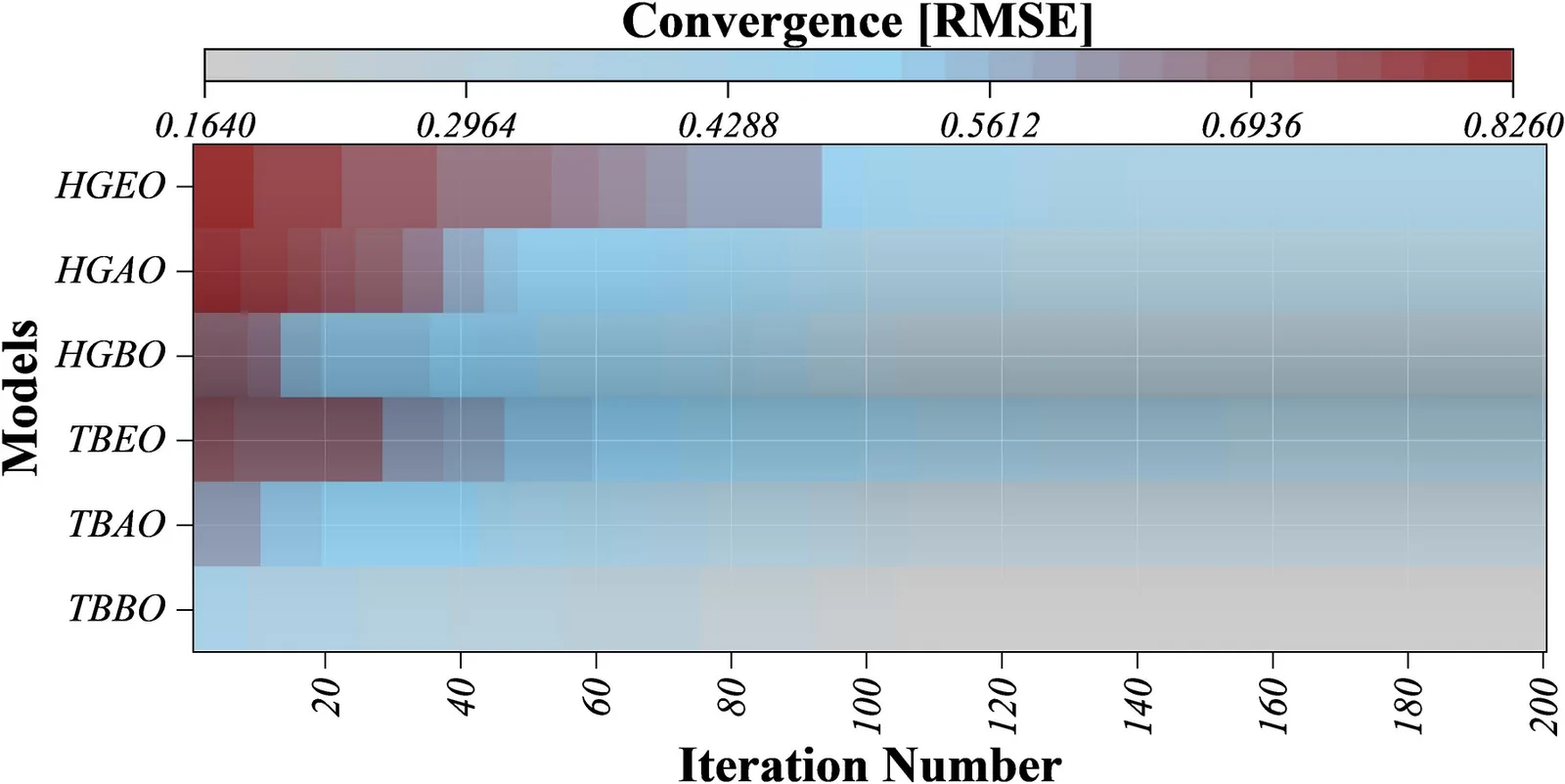
Heatmap for the convergence behavior of the models during optimization.

Figure 2 presents a convergence heatmap of RMSE over 200 optimization steps for all TabNet- and HGB-based hybrid models. It indicates visible distinctions between how they learn and how fast they computerize. RMSE constantly lowers during these iterations, showing that each optimizer is successfully lowering prediction error, which is a hint that the parameters have been set correctly. The TabNet-based hybrids (TBBO, TBAO, TBEO) converge swiftly, usually stabilizing at low RMSE values around 40 to 60 iterations. This quick improvement is probably because TabNet uses attention-driven mechanisms and bio-inspired optimization to make it easier to concentrate on important elements and cut down on unnecessary ones. The HGB-based hybrids (HGAO, HGEO, HGBO), on the other hand, show a more gradual and smooth convergence trend. Their approach suggests a more cautious exploration of the error surface, which appears to provide greater control over local fluctuations during optimization. All models reached stable minima before 150 iterations, showing that the algorithm is strong and the training is quick. From a pharmaceutical point of view, faster convergence means less computational overhead and faster model deployment. This is a big benefit when screening large molecular libraries or optimizing formulation parameters. So, the TabNet-based hybrids not only have high predictive accuracy, but they also work well with large amounts of data, which helps speed up drug discovery and solubility testing pipelines in complex thermodynamic conditions.

### Model performance comparison

Table 6 shows how well the baseline and hybrid models can predict, showing how metaheuristic optimization affects accuracy, generalization, and robustness. The hybrid architectures showed big improvements over their standalone versions on all metrics, which shows how useful optimizer-guided fine-tuning is. The TBBO configuration achieved the lowest RMSE (0.218) and one of the highest R² values (0.981) among the evaluated models during testing. However, the performance differences among several optimized configurations were relatively small across certain metrics, indicating that multiple optimizer–model combinations can achieve comparable predictive accuracy within the dataset.

The Model’s stability and ability to make accurate predictions are further supported by a low RSR (0.141) and a high U₉₅ (0.933). From a pharmaceutical point of view, this level of accuracy directly leads to more accurate solubility estimates, which are important for predicting bioavailability, improving formulations, and reducing the need for experimental testing. Even minor reductions in RMSE or MAPE can have a notable impact on dosage accuracy and product efficacy, which highlights the necessity for evaluating models across multiple metrics. While all the optimized variants performed admirably, TabNet-based hybrids generally outperformed their HGB-based counterparts, suggesting that attention-driven architectures adapt more effectively to metaheuristic optimization.

**Table 6:** Performance measurements of the models, evaluating their predicted accuracy and efficacy using essential statistical measures.

Process	Category	Models	Evaluation Metrics					
			RMSE	R^2^	MAPE	RSR	U95	FGE
Train	Single Models	Tabnet	0.447	0.951	23.459	0.253	1.232	0.105
		HGB	0.471	0.933	29.877	0.266	1.298	0.002
	Hybrid Models	TBBO	0.163	0.992	9.266	0.092	0.453	0.037
		TBAO	0.261	0.979	13.748	0.148	0.724	0.063
		TBEO	0.344	0.968	17.624	0.195	0.953	0.077
		HGBO	0.286	0.975	27.330	0.162	0.792	0.079
		HGAO	0.351	0.961	30.029	0.199	0.973	0.026
		HGEO	0.398	0.950	33.529	0.225	1.100	0.015
Validation	Single Models	Tabnet	0.289	0.910	23.126	0.302	0.801	0.093
		HGB	0.297	0.913	30.163	0.310	0.822	0.010
	Hybrid Models	TBBO	0.165	0.970	11.256	0.173	0.458	0.032
		TBAO	0.184	0.964	13.499	0.192	0.509	0.037
		TBEO	0.225	0.948	19.897	0.235	0.620	0.068
		HGBO	0.225	0.947	22.346	0.235	0.622	0.049
		HGAO	0.273	0.936	21.374	0.286	0.735	0.088
		HGEO	0.293	0.909	31.223	0.306	0.806	0.042
Test	Single Models	Tabnet	0.379	0.945	25.594	0.274	1.030	0.027
		HGB	0.406	0.924	34.096	0.293	1.103	0.082
	Hybrid Models	TBBO	0.182	0.983	16.014	0.131	0.503	0.022
		TBAO	0.278	0.963	21.049	0.201	0.753	0.039
		TBEO	0.329	0.963	26.421	0.238	0.885	0.035
		HGBO	0.247	0.972	27.449	0.178	0.672	0.049
		HGAO	0.286	0.960	23.389	0.207	0.783	0.030
		HGEO	0.332	0.947	35.256	0.240	0.920	0.074

### Correlation and variance consistency (Taylor diagram analysis)

**Fig. 3:**
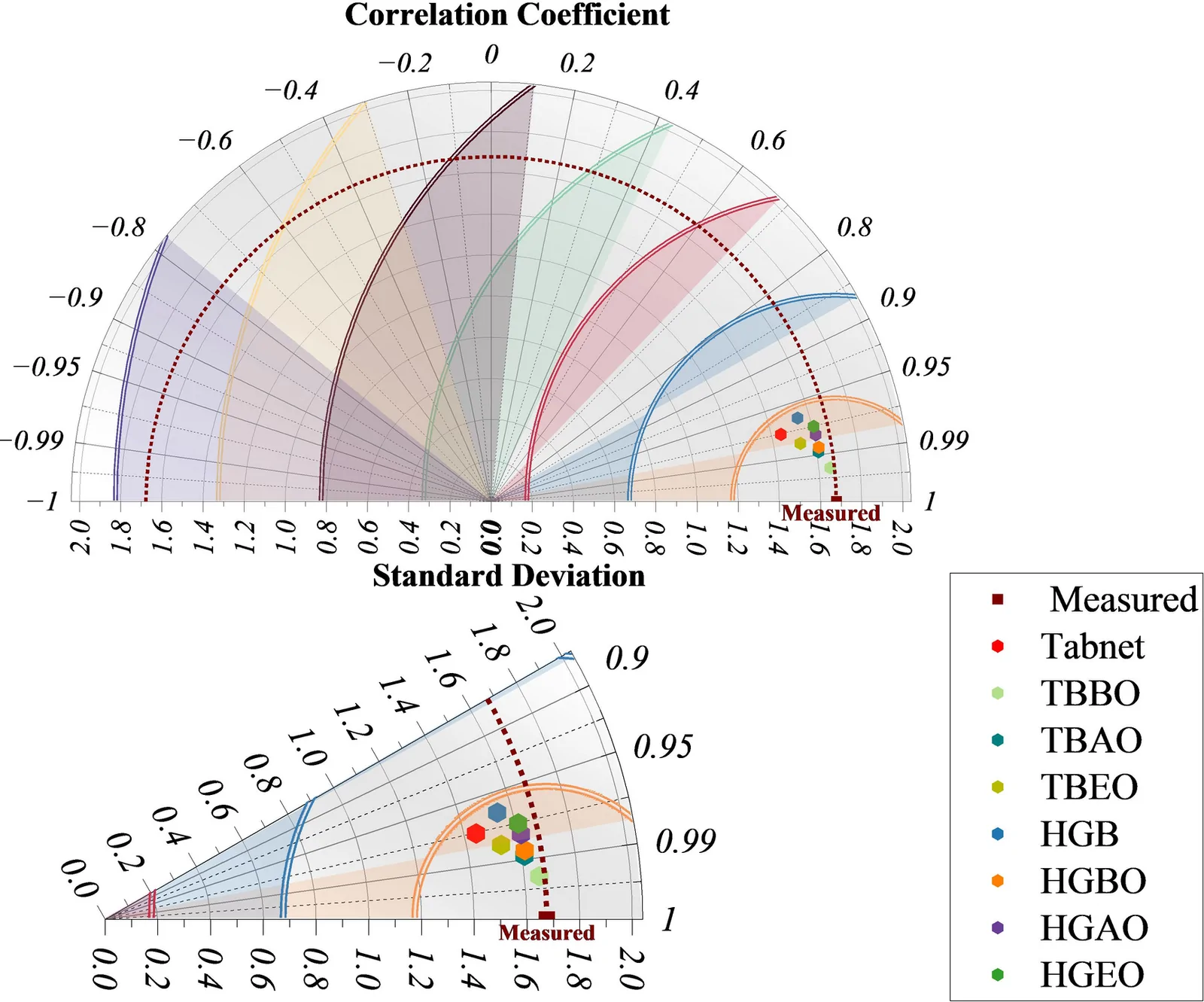
Taylor diagram for comparing model performance based on correlation and standard deviation.

Figure 3 presents the Taylor diagram, summarizing correlation, standard deviation, and RMS deviation of predicted versus experimental data. Hybrid models cluster tightly near the reference point, indicating strong correlation and accurate variance reproduction. TabNet-based hybrids (TBBO, TBAO, TBEO) achieved correlation coefficients near 0.99, demonstrating precise predictions and minimal error variation. HGB-based hybrids also show high correlations (*r* > 0.97) but with broader deviations, reflecting slightly higher variance. Overall, the Taylor diagram confirms strong fidelity and generalization, with TBBO providing the most stable and balanced predictions.

### Error distribution and predictive stability (residual analysis)

**Fig. 4:**
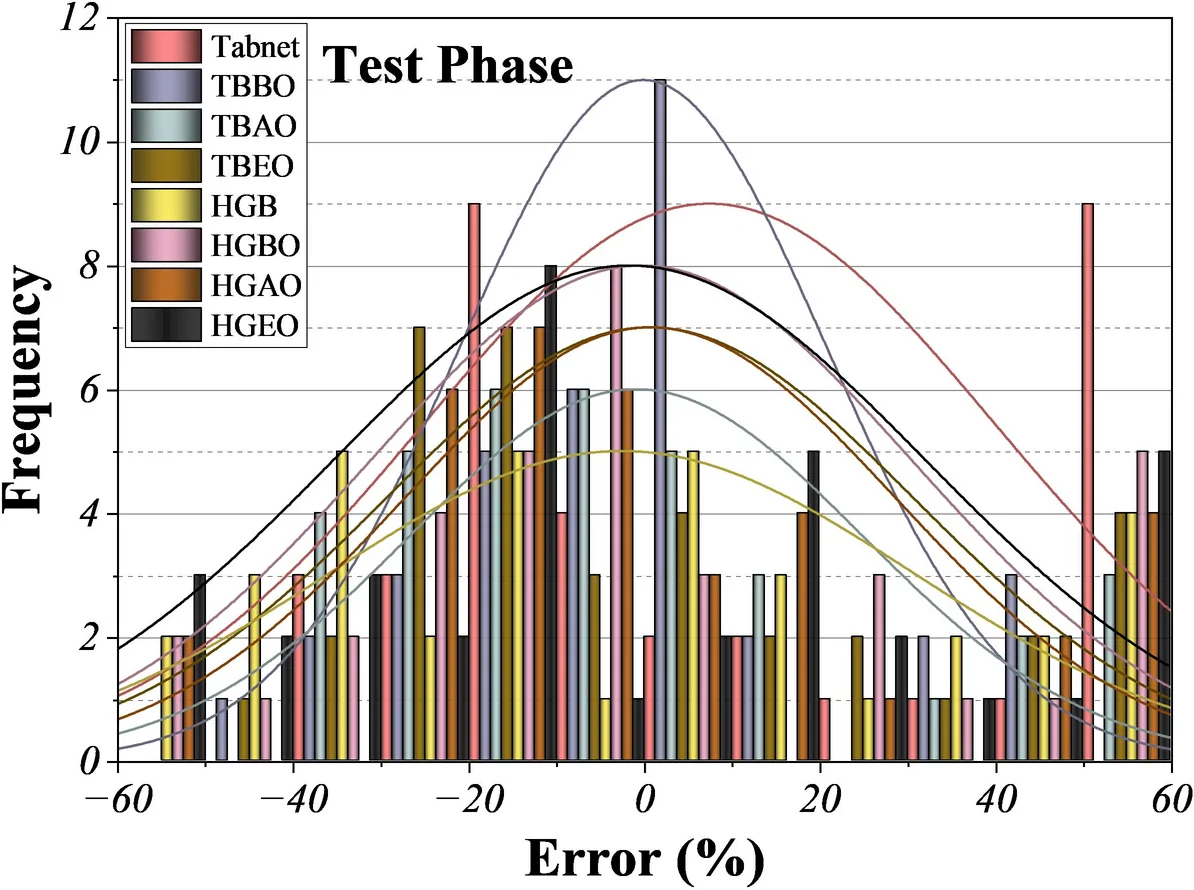
Histogram plot for error analysis, providing a general visualization of error distribution.

Figure 4 provides a residual-based perspective on model performance, illustrating how prediction errors are distributed around zero during the testing phase. This visualization complements numerical metrics by revealing each Model’s bias, variance, and generalization tendencies in predicting drug solubility under supercritical CO₂ conditions. The overall near-symmetric, Gaussian-like distribution across all models indicates that predictions are largely unbiased and centered around experimental values, confirming stable calibration. The HGB-based hybrids display narrower, smoother error curves with limited extreme deviations, signifying better generalization and consistent learning across diverse thermodynamic conditions. In contrast, the TabNet-based variants exhibit sharper but slightly broader peaks, reflecting strong local learning capacity with minor overfitting in specific parameter ranges. The application of bio-inspired optimizers, particularly the ALO, BOA, and EVO, visibly reduces high-magnitude residuals, leading to smoother, more symmetric error profiles. This distributional improvement highlights the effectiveness of optimization in refining model robustness and mitigating prediction bias. From a pharmaceutical modeling standpoint, the low and evenly distributed residuals demonstrate the model’s capability to reliably estimate solubility without systematic error. Such consistent predictive behavior underpins the practical potential of these hybrid frameworks to minimize experimental screening efforts and accelerate formulation design in SC-CO₂ systems.

### Feature influence and statistical sensitivity (Kruskal-Wallis analysis)

**Fig. 5:**
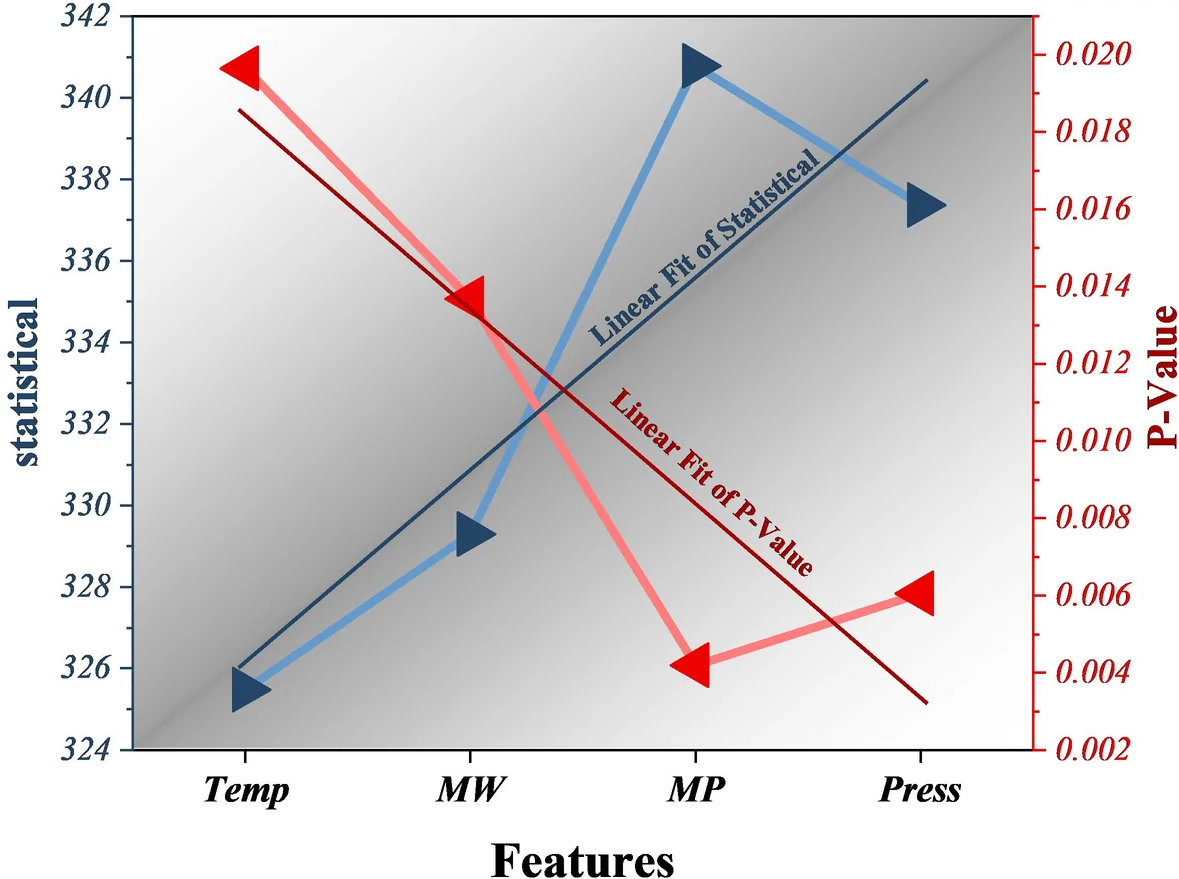
2Ys plot for Kruskal sensitivity analysis illustrating the relative impact of variables on model performance.

Figure 5 presents the 2Ys plot from the Kruskal-Wallis sensitivity analysis, offering a nonparametric assessment of how each input variable contributes to the predictive reliability of the hybrid solubility models. This method was employed because it does not assume data normality and instead ranks variables based on statistical significance, providing an unbiased measure of feature influence. The analysis reveals that temperature and pressure exert the greatest impact on model performance, supported by high statistical values and correspondingly low p-values. This dominance aligns with established thermodynamic understanding, where both parameters govern CO₂ density and solute diffusivity, directly shaping solubility behavior. In contrast, molecular weight and melting point show moderate but consistent contributions, reflecting their role in molecular cohesion and sublimation resistance. The inverse relationship between the Kruskal-Wallis statistic and p-value confirms that features with higher predictive relevance maintain stronger, statistically reliable effects. Such coherence demonstrates the robustness of the hybrid framework, as model-driven importance rankings align closely with physical solvation principles. In summary, this work affirms that the machine learning-optimization models not just make precise predication but also stay rooted in interpretable, experimentally consistent relations in SC-CO₂ systems, and validate their credibility for pharmaceutical solubility prediction and formulation design diagnosis.

### Multi-objective trade-off optimization (Pareto front evaluation)

**Fig. 6:**
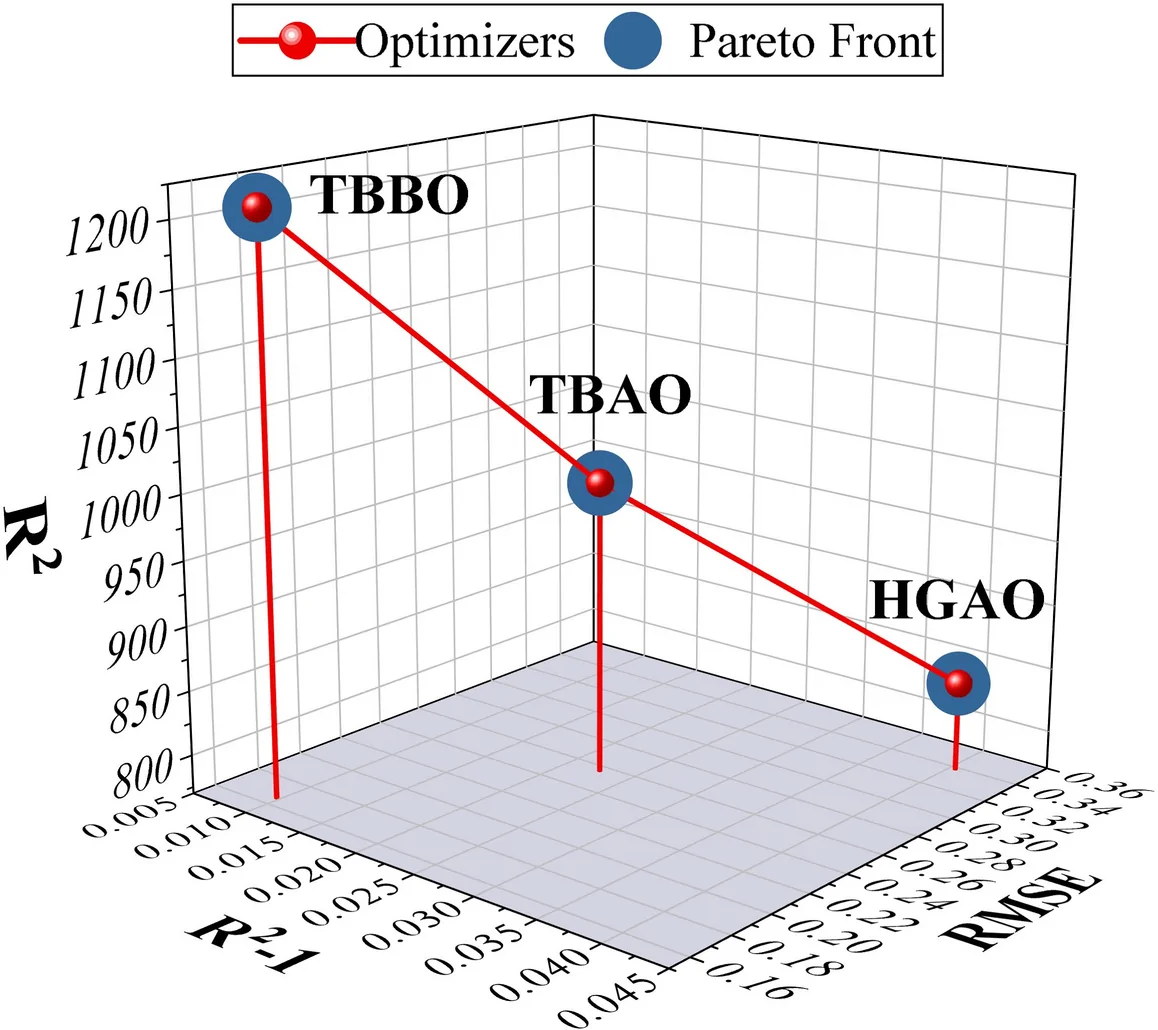
3D scatter plot for Pareto front illustrating the trade-offs between multiple objective functions in the optimization process.

Figure 6 indicates the resulting 3D Pareto front from the multi-objective optimization. It presents the trade-offs between other significant performance metrics: the root mean square error (RMSE), the coefficient of determination (R²), and the residual loss (R-1). This visualization allows for a comprehensive assessment of model performance, focusing on stability and generalization instead of a single accuracy metric. The position of the optimizers along the Pareto surface indicates various optimization dynamics. The Pareto front visualization illustrates the trade-offs between prediction accuracy and residual error across different optimizer–model configurations. Several hybrid models occupy regions of the Pareto surface corresponding to high R² values and relatively low RMSE. This distribution suggests that multiple optimization strategies can reach competitive performance levels under the evaluated objectives. The Pareto front therefore serves primarily as a visual diagnostic for examining trade-offs among metrics rather than as a definitive ranking tool.

TBAO approach strikes a good balance between various goals and ensuring that the system remains stable. HGAO, in contrast, adopts a safer search strategy, and thus error rates become a little higher but are sure to converge. The fact that there is a smooth Pareto front and no abrupt changes or unusual points indicates that the optimization process is robust and reliable. The data as a whole have a bearing that highlights the importance of a multi-objective approach in achieving balanced behavior and improved generalization.

### Computational efficiency and scalability assessment

Table 7 summarizes the computational runtime of the baseline models and the optimization-driven hybrid configurations across the complete modeling workflow. The reported runtimes primarily reflect the hyperparameter search procedure performed by the population-based optimizers rather than the intrinsic training time of the machine learning models themselves. Because these optimizers iteratively evaluate multiple candidate parameter configurations, the hybrid models require substantially longer runtimes than the baseline learners. The baseline models (TabNet and HGB) complete training in less than one second because they are executed using default or fixed hyperparameter settings. In contrast, the hybrid configurations require additional computational time due to the iterative exploration of the hyperparameter search space. Depending on the optimizer, the total runtime ranges from approximately 839 s to 1322 s, with TBEO exhibiting the longest runtime and HGAO the shortest among the optimized configurations. It should be emphasized that this computational cost corresponds to the model calibration stage, which is typically performed offline during model development. Once the optimal hyperparameters are identified, the final trained models can be executed for prediction with negligible computational cost comparable to the baseline learners. Therefore, the reported runtime reflects the additional effort required to explore the hyperparameter space rather than the operational cost of using the trained model. The inclusion of computational runtime statistics provides transparency regarding the computational demands of the optimization process and allows readers to assess the trade-offs between search effort and predictive performance within the evaluated modeling framework.

**Table 7:** Computation runtime of each model across all phases collectively (training, validation, and testing).

Models	Runtime
TBBO	1210.4 s
TBAO	996.5 s
TBEO	1322 s
Tabnet	0.93 s
HGBO	1018.4 s
HGAO	839 s
HGEO	1112 s
HGB	0.78 s

### Predictive interval validation (PIB)

Table 8 illustrates the predicted solubility of Predictive Interval Band (PIB) under varying thermodynamic conditions, revealing a clear interplay between temperature, pressure, molecular weight, and melting point. The Model indicates that solubility rises with increasing pressure due to enhanced CO₂ density, which strengthens solvent-polymer interactions and weakens polymer-polymer cohesion, an effect well established in supercritical thermodynamics. Temperature exerts a dual influence: moderate heating enhances solubility by improving molecular mobility and diffusion, whereas excessive heating reduces it by lowering CO₂ density and solvent power. Molecular structure also governs solubility behavior; lower- and intermediate-weight PIBs (≈ 315–586 g/mol) dissolve more readily because their reduced cohesive energy facilitates penetration of CO₂ molecules, while very high molecular weights (> 900 g/mol) exhibit diffusion limitations and lower solubility. Notably, the Model reproduces nonlinear and coupled effects; for instance, pressure-driven gains diminish at extreme temperatures, highlighting its physical interpretability beyond statistical fitting. These patterns align closely with established experimental trends in SC-CO₂ systems, confirming model reliability.

**Table 8:** Predicted solubility values of pharmaceutical compounds in supercritical CO₂ using the optimized hybrid model within the Predictive Interval Band (PIB).

Temperature (K)	Molecular weight (g/mol)	Melting point (C)	Pressure (MPa)	Prediction
338	157.1	190	15	0.37003
318	586.18	146	18	0.580024
318	24	804.03	128	0.156035
328	315.71	239	180	1.390003
318	926.09	155	18	0.75002
328	21	822.95	185	1.596997
318	157.1	190	24	4.289925
328	157.1	190	18	1.390003
328	393.44	230	27	1.237007
328	586.18	146	27	2.939961
338	385.37	236	240	0.433028
318	15	270.21	231	0.826018
318	393.44	230	21	0.73002
318	586.18	146	27	1.569998
328	393.44	230	18	0.36803
308	926.09	155	24	1.280005
318	157.1	190	15	0.800018
308	337.45	163.5	18	1.552998
308	27	822.95	185	2.170982
338	393.44	230	15	0.119036
338	30	804.03	128	0.235033
338	315.71	239	240	2.559971
318	385.37	236	120	0.034039
328	12	822.95	185	0.238033
318	157.1	190	12	0.37003
308	21	822.95	185	1.636996
323	20	914.172	183	0.324031
328	315.71	239	210	1.909989
328	586.18	146	21	1.380003
338	18	804.03	128	0.096037
308	315.71	239	210	2.029986
318	385.37	236	270	0.413028
318	12	822.95	185	0.482027
338	27	270.21	231	2.712967
318	157.1	190	18	1.300005
338	315.71	239	120	0.110037
328	157.1	190	27	6.579865
338	18	822.95	185	1.09801
318	30	822.95	185	2.765966
328	15	804.03	128	0.076037
318	385.37	236	300	0.493026
318	315.71	239	180	1.539999
318	926.09	155	15	0.530025
308	157.1	190	15	0.870016
328	315.71	239	120	0.200034
338	21	804.03	128	0.184035
338	385.37	236	150	0.083037
328	337.45	163.5	12	0.120036
338	385.37	236	120	0.019039
308	393.44	230	12	0.279032
328	20	914.172	183	0.381029
338	586.18	146	24	3.199954
318	315.71	239	120	0.690021
318	926.09	155	12	0.270032
338	586.18	146	21	1.809991
308	18	822.95	185	1.304005
338	393.44	230	24	1.049012
318	315.71	239	300	3.75994
308	586.18	146	21	0.480027
328	22.5	914.172	183	0.407029
308	926.09	155	12	0.450027
308	24	822.95	185	1.84399
328	315.71	239	240	2.519973
318	926.09	155	21	1.020012
318	157.1	190	18	1.300005
338	157.1	190	21	3.919935
318	157.1	190	15	0.800018
308	337.45	163.5	12	0.770019
318	586.18	146	21	0.890016
308	926.09	155	18	0.920015
318	157.1	190	21	2.719967

### Thermodynamic interaction mapping (temperature-pressure coupling analysis)

**Fig. 7:**
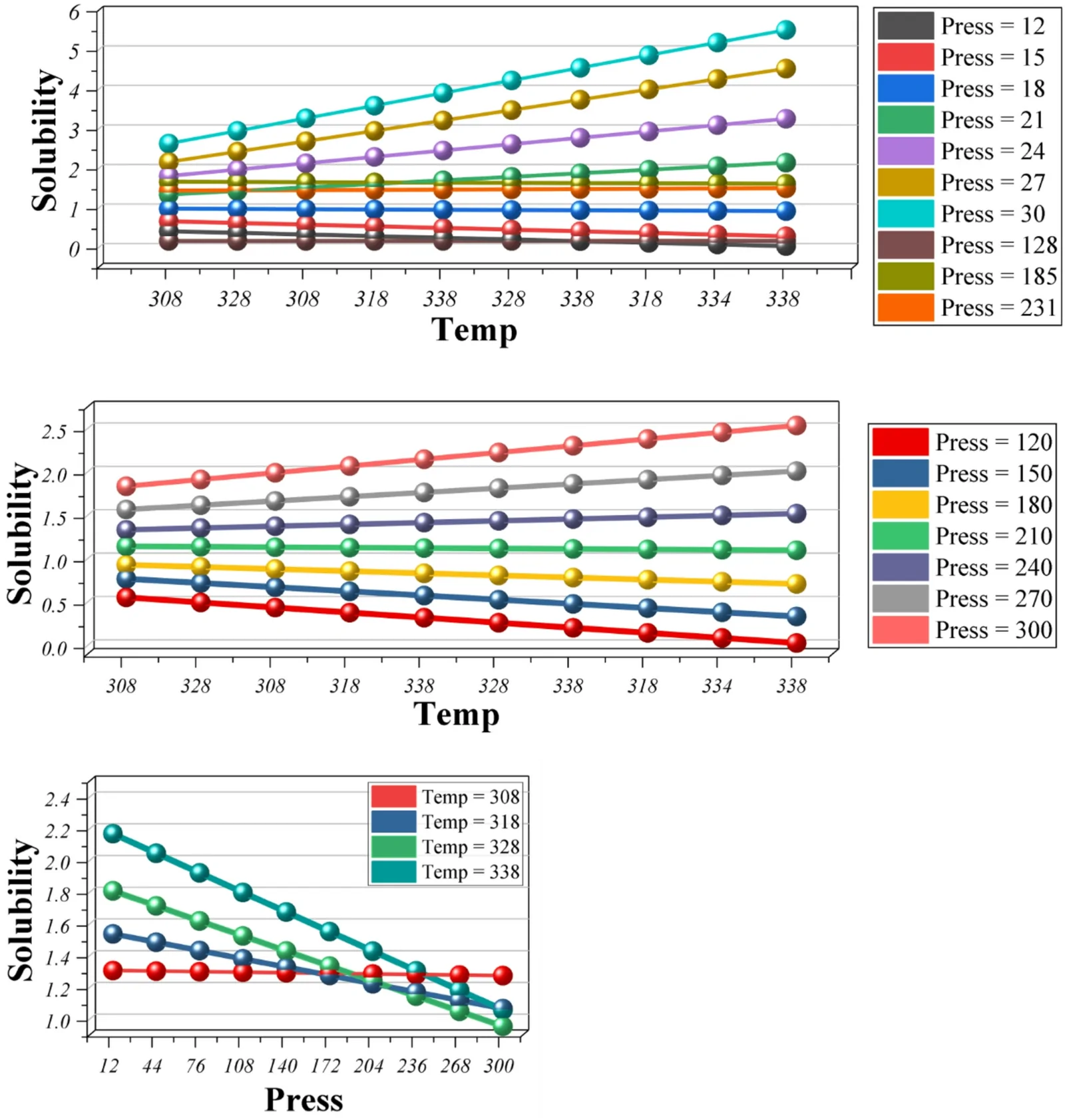
3D line-symbol for showing the tendency and variation of features across different conditions.

Figure 7 depicts the multidimensional interplay between temperature and pressure on the predicted solubility. As temperature increases, solubility generally rises at fixed pressures, a trend driven by enhanced molecular motion and reduced cohesive energy within the polymer matrix. However, this effect is moderated at very high pressures, where elevated CO₂ density limits further solvation improvement. In contrast, when temperature is held constant, an inverse relationship between solubility and pressure emerges; this stems from the nonlinear compressibility of supercritical CO₂. For lower pressures, the CO₂ compresses normally, but as density approaches saturation, the compressibility decreases. The Model effectively reproduces the subtle curvature and crossover characteristics intrinsic to these systems, which effectively unveil nonlinear thermophysical connections that are normally missed by conventional linear regression approaches. These coupled dynamics validate that the Model conforms to known supercritical thermodynamics, which indicates that it can be interpreted physically and used to make reliable predictions.

### Discussion and practical applications

The proposed hybrid architecture, integrating TabNet and HGB with three metaheuristic optimizers (ALO, EVO, and BOA), demonstrates the capability of data-driven models to capture nonlinear relationships governing drug solubility in supercritical CO₂ systems. Among the tested configurations, the TabNet–BOA combination (TBBO) achieved the highest predictive performance within the evaluated dataset, reaching R² = 0.981 and RMSE = 0.218. These results indicate that optimizer-assisted hyperparameter tuning can improve predictive consistency for structured thermodynamic data. However, it should be noted that such improvements may also reflect the influence of the optimization search process and the characteristics of the available dataset rather than an inherent superiority of a particular optimizer. The observed convergence patterns of the hybrid models suggest that different optimizers explore the hyperparameter space in distinct ways. For example, BOA and EVO tended to reach stable solutions more rapidly than some alternative configurations, while ALO demonstrated a more gradual search trajectory. Although faster convergence may indicate efficient exploration of the optimization landscape, it should not be interpreted as definitive evidence of better model generalization. Convergence speed can also be influenced by the structure of the search space, initialization conditions, or the relatively low dimensionality of the model parameter set.

Similarly, the smooth Pareto fronts obtained during multi-objective optimization provide a useful visualization of the trade-offs among accuracy, residual error, and other performance metrics. Nevertheless, such patterns should be interpreted cautiously, as smooth Pareto structures may arise from stable optimization dynamics rather than uniquely superior predictive models. For this reason, model evaluation in the present study relies primarily on quantitative statistical indicators and cross-validation results rather than qualitative visual diagnostics alone. The hybrid models were also able to reproduce general thermodynamic trends consistent with established supercritical solubility behavior. In particular, solubility generally increased with temperature under moderate pressures and tended to exhibit saturation behavior at higher CO₂ densities. These patterns align with widely reported observations in supercritical fluid systems, where temperature influences both molecular diffusivity and solvent density. The Kruskal–Wallis sensitivity analysis further identified temperature and pressure as the most statistically influential variables, while molecular weight and melting point contributed secondary effects related to intermolecular cohesion and crystal stability. The correspondence between these feature importance patterns and established thermodynamic principles supports the physical plausibility of the learned relationships.

- *Limitation of the study*: At the same time, several limitations should be acknowledged. The dataset used in this study, while experimentally curated, contains approximately 350 observations and only four primary descriptors. Although cross-validation and statistical testing were applied to mitigate overfitting, models trained on datasets of this scale remain sensitive to potential hyperparameter tuning bias or dataset-specific correlations. Consequently, the reported predictive performance should be interpreted primarily within the thermodynamic domain represented by the compiled experimental data.
- *Future research suggestion*: Future research may address these limitations by incorporating larger and more diverse datasets, integrating physics-informed constraints or hybrid thermodynamic models, and validating performance on independent experimental datasets. The inclusion of advanced molecular descriptors and the exploration of transfer learning strategies may further improve model generalizability. Additionally, extending the framework to multicomponent systems and dynamic solubility behavior represents a valuable direction for future work.
- *Application perspective*: From an application perspective, the proposed framework may serve as a computational tool for preliminary solubility screening and formulation analysis in supercritical CO₂ environments. By providing rapid predictions across a range of temperature and pressure conditions, such models may assist in guiding experimental design and reducing the number of required laboratory trials. Potential applications include early-stage drug formulation studies, optimization of supercritical extraction processes, and evaluation of operating conditions in CO₂-based pharmaceutical processing. The integration of uncertainty-related metrics and cross-validation procedures further supports the framework’s use as a decision-support tool rather than a replacement for experimental validation.
- *Advantages of the study*: The proposed framework offers several advantages. It combines interpretable and ensemble learning methods to capture nonlinear solubility behavior while maintaining computational efficiency. The integration of metaheuristic optimization improves parameter tuning, and the use of multiple validation techniques enhances statistical reliability. In addition, the framework operates on a limited set of experimentally accessible variables, making it practical for real-world applications where extensive molecular descriptors may not be available. However, certain disadvantages should also be acknowledged. The reliance on a relatively small dataset may limit the robustness of the learned relationships. Furthermore, the absence of explicit molecular descriptors restricts the model’s ability to capture detailed chemical interactions. The use of metaheuristic optimization also increases computational cost compared to conventional tuning approaches. These factors indicate that while the framework is effective within the studied domain, its performance may vary under different data conditions.

## Summary and conclusion

This study proposed a hybrid learning framework that bridges data-driven prediction and thermodynamic understanding for modeling drug solubility in supercritical carbon dioxide (SC-CO₂). By integrating the interpretable feature learning of TabNet, the gradient stability of HGB, and the adaptive search dynamics of three metaheuristic optimizers, ALO, EVO, and BOA, the framework establishes a transparent and robust alternative to conventional black-box or semi-empirical approaches. Its novelty lies not merely in algorithmic fusion but in creating a balanced system capable of achieving interpretability, generalizability, and predictive precision within a single modeling pipeline. Quantitative analyses demonstrated that the BOA-optimized hybrid achieved the most stable trade-off between accuracy and uncertainty, reaching an average $\:{R}^{2}$≈0.98 and maintaining narrow prediction intervals (< 5%) across the 366 experimental samples. The results demonstrate that the model captures statistical relationships between temperature, pressure, and molecular descriptors that are consistent with widely reported thermodynamic trends in SC-CO₂ solubility systems.

The Kruskal-Wallis test further reinforced these findings by highlighting temperature and pressure as statistically significant factors, in line with established solubility theory. Taken together, these findings indicate that the framework accurately captures real physicochemical relationships, rather than just statistical anomalies. This research connects artificial intelligence and physical chemistry by turning complicated solubility behavior into easy-to-understand predictive structures. The Model’s dependability, demonstrated through K-fold cross-validation and bootstrap uncertainty analysis, establishes it as a robust decision-support instrument for pharmaceutical process design, especially in supercritical extraction and formulation optimization. Building on the principles of green chemistry, this framework supports solvent-efficient process development and advances sustainable drug formulation, particularly within high-pressure CO₂ settings. While it demonstrates considerable versatility, there is potential for further expansion, such as application to multicomponent mixtures, mixed-solvent systems, and investigations into time-dependent solubility kinetics.

Despite these encouraging results, several limitations should be considered. The dataset used in this study contains approximately 350 experimental observations and includes only four primary descriptors. Although cross-validation and statistical evaluation were applied to mitigate overfitting, models trained on datasets of this scale remain sensitive to dataset composition and hyperparameter optimization strategies. Furthermore, the present validation relies primarily on internal cross-validation rather than independent external datasets, which limits the ability to fully assess model generalization across broader chemical domains. For these reasons, the proposed framework should be interpreted as a data-driven predictive approach that complements, rather than replaces, traditional thermodynamic or semi-empirical modeling techniques. Its primary value lies in its ability to rapidly explore potential relationships within available experimental data and to support preliminary analysis of solubility behavior under different operating conditions.

## Supplementary Information

Below is the link to the electronic supplementary material.

Supplementary Material 1.

## Data Availability

The dataset used in this study, has been provided as supplementary material in Excel format.

## Abbreviations

APIs: Active pharmaceutical ingredients
Tabnet: Interpretable deep learning
ALO: Attack-leave optimizer
BOA: Botox optimization algorithm
U95: Uncertainty at 95% confidence level
R^2^: Coefficient of determination
MAPE: Mean absolute percentage error
SC-CO₂: Supercritical carbon dioxide
HGB: Histogram-based gradient boosting
EVO: Energy valley optimizer
FGE: Fractional gross error
RSR: Ratio of rmse to the standard deviation of observations
RMSE: Root mean squared error
